# Patient‐reported disruptions to cancer care during the COVID‐19 pandemic: A national cross‐sectional study

**DOI:** 10.1002/cam4.5270

**Published:** 2022-10-07

**Authors:** Jacob J. Lang, Aparna Narendrula, Sharanya Iyer, Kristine Zanotti, Puneet Sindhwani, Elias Mossialos, Obi Ekwenna

**Affiliations:** ^1^ University of Toledo College of Medicine and Life Sciences Toledo Ohio USA; ^2^ Case Western Reserve University School of Medicine Cleveland Ohio USA; ^3^ Department of OB/GYN‐Gynecological Oncology University Hospitals Cleveland Medical Center Cleveland Ohio USA; ^4^ Population and Cancer Prevention Program Case Comprehensive Cancer Center Cleveland Ohio USA; ^5^ Department of Urology and Transplantation University of Toledo College of Medicine and Life Sciences Toledo Ohio USA; ^6^ Department of Health Policy London School of Economics and Political Science London UK; ^7^ Institute of Global Health Innovation, Imperial College London London UK

## Abstract

**Background:**

The aim of this study is to evaluate the extent and associations with patient‐reported disruptions to cancer treatment and cancer‐related care during the COVID‐19 pandemic utilizing nationally representative data.

**Methods:**

This analysis uses data from the 2020 National Health Interview Survey (NHIS), an annual, cross‐sectional survey of US adults. Adults (age >18) who reported requiring current cancer treatment or other cancer‐related medical care in the second half of 2020 were included. Estimated proportions of patients with self‐reported changes, delays, or cancelations to cancer treatment or other cancer care due to the COVID‐19 pandemic were calculated using sampling weights and associations with sociodemographic and other health‐related variables were analyzed.

**Results:**

In total, 574 (sample‐weighted estimate of 2,867,326) adults reported requiring cancer treatment and/or other cancer care since the start of the COVID‐19 pandemic. An estimated 32.1% reported any change, delay, or cancelation. On sample‐weighted univariable analysis, patients who were younger, female, had one or fewer comorbidities, and uninsured were significantly more likely to report disruptions. On sample‐weighted, multivariable analysis, patients who were younger and female remained significant predictors. Nearly 90% of patients included in the study reported virtual appointment use. Patients reporting disruptions were also significantly more likely to report feelings of anxiety.

**Conclusions:**

An estimated 1/3 of patients experienced disruptions to cancer care due to the COVID‐19 pandemic. Patients experiencing disruptions in care were more likely to be female or younger which may reflect risk stratification strategies in the early stages of the pandemic, and also had higher rates of anxiety. The longitudinal impact of these disruptions on outcomes merits further study.

## Graphical abstract

In an analysis of National Health Interview Survey (NHIS) data from 2020, we found nearly 1/3 of patients reportedly requiring cancer treatment or other cancer care experienced disruptions due to the COVID‐19 pandemic. Patients experiencing disruptions were significantly more likely to be younger or female, as well as reported higher rates of anxiety. The long term impact of these care disruptions on outcomes merits further study.

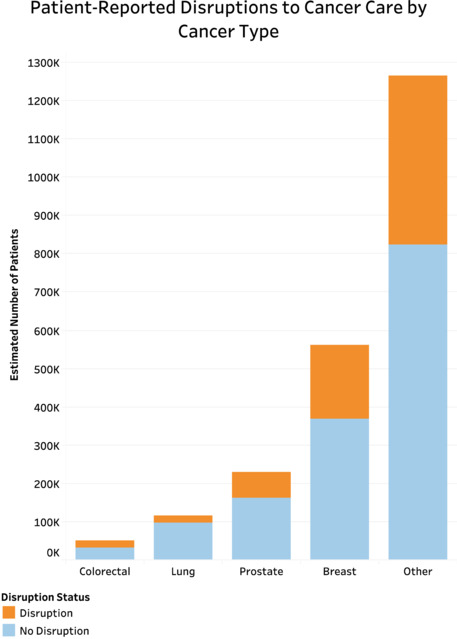

## 1 INTRODUCTION

The COVID‐19 pandemic has been immensely disruptive to the healthcare system in the United States, permeating nearly every aspect of medical care.^1^ Regional disease‐mitigation efforts, closure of healthcare facilities, increased overall burden to the healthcare system, and self‐delays in care due to concern for COVID‐19 have all contributed to disruptions in care.^2^, ^3^ Additionally, care delays have been more pronounced in individuals with disabilities and multiple comorbidities.^2^ While these patient populations are at risk for severe COVID‐19 infection, they also are more likely to be impacted by care delay due to their increased medical needs. This is likely especially prominent in individuals receiving cancer care.^4^

Due to the recent and evolving nature of COVID‐19, however, study of the impact of the pandemic on cancer care is limited. One survey of breast cancer survivors showed that 44% of surveyed patients self‐reported treatment delay in the first few weeks of the pandemic.^5^ These delays were present in all aspects of care, including surgery, imaging, lab testing, genetic counseling, chemotherapy, and routine follow‐up.^5^ Another study demonstrated that in the United Kingdom, factors including fear of the pandemic and changes to healthcare policy that shifted nearly all resources to handling COVID‐19 led to declines in cancer screening and therefore decreased diagnosis for certain types of cancer.^6^ Other factors that may impact care include lockdowns, financial issues, travel, healthcare supply shortages, healthcare worker shortages, and delays in non‐emergent surgical procedures.^7^ While some of these measures may protect oncology patients from contracting COVID‐19, their impact on patient access to cancer care during the pandemic has not been fully understood.^7^

This study aims to characterize and analyze national patient‐reported delays in cancer care in the US due to the COVID‐19 pandemic for all types of cancer. We employ nationally representative data from the National Health Interview Survey (NHIS) to identify self‐reported disruptions in cancer care and their relationships across sociodemographic, geographic, and other care‐related groups to gain a better understanding of how the COVID‐19 pandemic has impacted cancer care.

## 2 METHODS

### 2.1 Data source and study sample

We extracted NHIS data from the Sample Adult Interview for 2020. The NHIS is an annual, cross‐sectional survey conducted by the National Center for Health Statistics (NCHS), a division of the Centers for Disease Control and Prevention (CDC).^8^ The survey collects health information and resource utilization of the civilian noninstitutionalized US population. The NHIS Sample Adult Interview provides health and demographic information for a large, nationally representative sample of US adults. The sample‐weighted estimates reported in this study were calculated using weighting procedures specified by the NHIS. During the third and fourth quarter of survey collection in 2020, four variables assessing patient oncology treatment or other cancer care status were introduced to assess the impact of the COVID‐19 pandemic on oncology care. Consent was obtained through the agency conducting NHIS surveys, as noted in CDC documentation.^8^ As NHIS data are de‐identified and publicly available, the study did not constitute human subjects research and was determined to be exempt from Institutional Review Board (IRB) approval.

**FIGURE 1:**
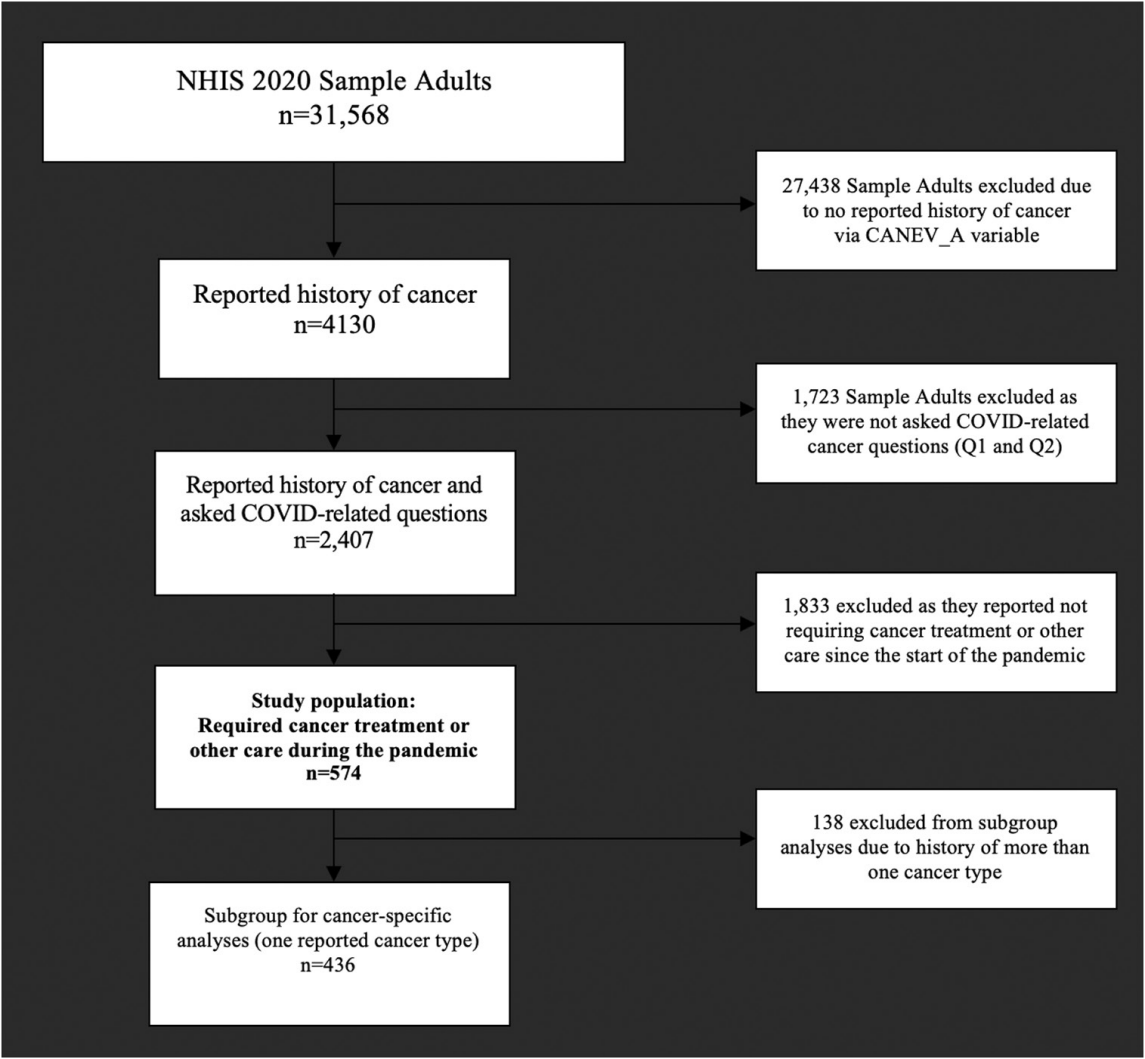
Flow diagram for study population inclusion. If variable‐specific subgroup populations are missing data, the reported number included is listed in Table 1.

Sample adults (age > 18) who reported “Yes” to a history of cancer requiring current treatment including “surgery, radiation therapy, chemotherapy, bone marrow transplants, stem cell transplants, or hormone therapy”, or adults with history of cancer requiring current other medical care related to their cancer including “lab visits, imaging, monitoring visits, rehabilitation, physical therapy, care for side‐effects, or visits with medical specialist”, in the third or fourth quarter of 2020 were included. Those with unknown cancer treatment and other cancer care status during the pandemic were excluded (Figure 1).

### 2.2 Key variables

#### 2.2.1 Cancer care

Responses to two questions assessing impact of COVID‐19 on cancer treatment or other cancer care were used as the main outcome variables: (1) “Were any of your treatments for cancer changed, delayed, or cancelled because of the coronavirus pandemic?”, (2) “Was any of this other medical care related to your cancer changed, delayed, or cancelled because of the coronavirus pandemic?” Responses were converted to a binary variable with those reporting “Yes” to either variable categorized as one group and those with “No” or “Do not Know” (*n* = 1) responses to both questions categorized into the other group.

#### 2.2.2 Telehealth use

Data on reported virtual appointment use due to COVID‐19 were also included. Sample adults responded to the following question: Were any of your appointments done by video or by phone because of reasons related to the coronavirus pandemic?

#### 2.2.3 Demographic, socioeconomic, and other health‐related variables

Self‐reported demographic variables included age, sex, race (classified as Black, White, or other due to limited sample variability), Hispanic ethnicity, urban–rural classification, region, and socioeconomic variables including insurance status (private, government‐sponsored, or uninsured) and household income. Reported history of cancer type was also include, and patients were grouped into breast, prostate, lung, colon & rectal, and other cancer, and a sub‐analysis was performed on patients reporting history of only one cancer type. Reported history of comorbidities associated with worse COVID‐19 outcomes including hypertension, hypercholesterolemia, cardiovascular disease, asthma, diabetes, chronic obstructive pulmonary disease, and kidney disease were also collected and categorized.^9^, ^10^, ^11^ Reported immunosuppressed status, both by medication and underlying health conditions, were also extracted and analyzed. Reported frequencies of feelings of anxiety, nervousness, or worry, as well as depression, were sorted into two binary variables and analyzed, with those reporting weekly or daily feelings as positive and those reporting never, a few times a year, or monthly as negative for both variables.

### 2.3 Statistical analysis

As the NHIS is a complex, multistage probability sample that uses stratification, clustering, and oversampling of some populations, all data were adjusted using sample weights and specific variables to account for stratification and other survey characteristics using the Stata svy command.^12^, ^13^, ^14^ Sample adult weights were proportionally inflated within categories of demographic variables until marginals matched or approximated population counts across all dimensions to match population counts, including by adjusting for race/ethnicity.^15^ The primary outcome of our study was sample‐weighted estimated proportion of patients reporting change, delay, or cancelation of cancer treatment or other cancer care due to the COVID‐19 pandemic. This estimate reflects application of weights across within survey sub‐stratification to account for under sampling of some populations. Chi‐square testing was used to compare sample‐weighted estimated proportions across variable groups. Sample‐weighted univariable logistic regression analysis was utilized to assess potential variable associations with change, delay, or cancelations to cancer care during the COVID‐19 pandemic. Sample‐weighted multivariable logistic regression analysis was used to create a model adjusted for race/ethnicity, age, sex, insurance status, urban–rural classification, income, and number of comorbidities. Sample‐weighted logistic regression estimates were utilized as the NHIS survey was structured with consideration of projection of population totals with sample‐weighting from its inception, thus it was determined the most robust method of estimating associations. Unweighted univariable and multivariable logistic regression estimates were obtained and reported for completeness, but conclusions were made from weighted estimates. Sample adults with missing variable data were excluded from variable‐specific analysis and the included variable subpopulations are listed in Table 1. Significance was assessed using two‐sided *p* values, with *p* < 0.05 considered statistically significant. All analyses were performed in Stata Software (Version 14.2; StataCorp LLC) and data visualization was performed in Tableau Software (Tableau Software LLC).

**TABLE 1:** Characteristics of patients reportedly requiring cancer treatment or other cancer care during the COVID‐19 pandemic

Characteristic	Sample data (*n* = 574; sample %)	Population estimate (sample‐weighted) (*N* = 2,867,326) (%)
Age (years)		
<65	208 (36.2)	1,243,438 (43.4)
65–74	201 (35.0)	925,560 (32.3)
75 or greater	165 (28.7)	697,328 (24.3)
Sex		
Female	326 (56.8)	1,655,381 (57.7)
Male	248 (43.2)	1,211,945 (42.3)
Race		
White	501 (87.3)	2,377,069 (82.9)
Black	31 (5.4)	182,058 (6.4)
Other	42 (7.3)	308,199 (10.8)
Hispanic		
Yes	24 (4.2)	184,588 (6.4)
No	550 (95.8)	2,682,738 (93.6)
Urban–Rural Classification		
Large central metropolitan	149 (26.0)	700,098 (24.4)
Large fringe metropolitan	128 (22.2)	697,732 (24.3)
Medium and small metropolitan	191 (33.3)	918,388 (32.0)
Nonmetropolitan	106 (18.5)	551,108 (19.2)
Region		
Northeast	98 (17.1)	514,890 (18.0)
Midwest	144 (25.1)	669,599 (23.4)
South	177 (13.4)	962,695 (33.6)
West	155 (27.0)	720,141 (25.1)
Household Income		
Less than 40,000	185 (32.2)	895,139 (31.2)
40,000–79,999	179 (31.2)	796,149 (27.8)
80,000 or greater	210 (36.6)	1,176,037 (41.0)
Insurance status		
Government	244 (42.5)	1,140,380 (39.8)
Private	319 (55.6)	1,626,760 (56.7)
Uninsured	11 (1.9)	100,186 (3.5)
Cancer type(s)^a^		
Bladder	14 (2.4)	58,103 (2.0)
Blood	7 (1.2)	46,651 (1.6)
Bone	5 (0.9)	26,643 (0.9)
Brain	5 (0.9)	36,686 (1.3)
Breast	138 (24.0)	748,858 (26.1)
Cervical	7 (1.2)	29,874 (1.8)^b^
Colon	25 (4.4)	110,736 (3.9)
Esophageal	2 (0.3)	10,528 (0.4)
Gallbladder	1 (0.2)	7943 (0.3)
Larynx‐trachea	1 (0.2)	4467 (0.2)
Leukemia	14 (2.4)	77,215 (2.7)
Liver	11 (1.9)	44,828 (1.6)
Lung	41 (7.1)	202,822 (7.1)
Lymphoma	24 (4.2)	127,144 (4.4)
Melanoma	29 (5.1)	140,568 (4.9)
Mouth, tongue, or lip	1 (0.2)	2311 (0.1)
Ovary	12 (2.1)	59,103 (3.6)^b^
Pancreatic	5 (0.9)	17,057 (0.6)
Prostate	81 (14.1)	384,965 (31.8)^c^
Rectal	4 (0.7)	13,529 (0.5)
Skin melanoma	50 (8.7)	232,237 (8.1)
Skin non‐melanoma	123 (21.4)	534,562 (18.6)
Skin (unknown)	23 (4.0)	118,049 (4.1)
Stomach	1 (0.2)	625 (0.02)
Throat‐pharynx	4 (0.7)	13,631 (0.5)
Thyroid	14 (2.4)	42,487 (1.5)
Uterine	19 (3.3)	102,699 (6.2)^c^
Head and neck	6 (1.0)	20,410 (0.7)
Colorectal	29 (5.1)	124,265 (4.3)
Other	67 (11.7)	402,502 (14.0)
Cancer subgroups	(*n* = 436)	(*N* = 2,222,992)
Breast	101 (23.2)	562,036 (25.3)
Prostate	50 (11.5)	230,158 (10.4)
Lung	21 (4.8)	115,338 (5.2)
Colon and Rectal	11 (2.5)	50,680 (2.3)
Other	253 (44.1)	1,264,780 (56.9)
Comorbidities		
Hypertension	311 (54.2)	1,460,355 (50.9)
High cholesterol	247 (43.0)	1,173,512 (40.9)
Cardiovascular disease (Coronary artery disease, angina, myocardial infarction, stroke)	78 (13.6)	328,200 (11.5)
Asthma	108 (18.8)	491,060 (17.1)
Diabetes	85 (14.8)	404,827 (14.1)
Chronic obstructive pulmonary disease	93 (16.2)	442,471 (15.4)
Weak or failing kidneys	63 (11.0)	336,663 (11.7)
No. of comorbidities		
Zero or one	284 (49.5)	1,489,518 (52.0)
Two or more	290 (50.5)	1,377,808 (48.0)
Weakened immune system due to prescriptions		
Yes	115 (20.0)	542,143 (18.9)
No	454 (79.1)	2,292,271 (80.0)
Do not Know	5 (0.9)	32,912 (1.2)
Weakened immune system due to health condition		
Yes	121 (21.1)	577,587 (20.1)
No	447 (77.9)	2,238,316 (78.1)
Do not Know	6 (1.0)	51,424 (1.8)
Worried, nervous, or anxious^d^		
Daily	101 (17.6)	508,105 (17.7)
Weekly	74 (12.9)	368,227 (12.8)
Monthly	44 (7.7)	214,583 (7.5)
A few times a year	189 (32.9)	990,786 (34.6)
Never	157 (27.4)	746,244 (26.0)
Do not Know	6 (1.0)	28,567 (1.0)
Depressed^d^		
Daily	40 (7.0)	205,890 (7.2)
Weekly	43 (7.5)	196,030 (6.8)
Monthly	52 (9.1)	267,172 (9.4)
A few times a year	149 (26.0)	736,641 (25.7)
Never	281 (49.0)	1,428,363 (49.8)
Do not Know	5 (0.9)	16,443 (0.6)
Reported care required		
Treatment	140 (24.4)	708,709 (24.7)
Other cancer care	286 (49.8)	1,370,329 (47.8)
Both	148 (25.8)	788,288 (27.5)
Virtual appointment related to COVID‐19	(*n* = 331)	(*N* = 1,600,587)
Yes	291 (87.9)	1,406,845 (87.9)
No	40 (12.1)	193,741 (12.1)

^a^ Patients could report history of up to 3 cancer types, so the total is greater than 100% percent. Additionally, cervical, ovarian, and prostate cancers are sex‐specific, and therefore the proportion of respondents are taken out of their respective sex subpopulation, listed below. The survey does not mention for which cancer patients are currently receiving care.

^b^ Subpopulation is all included females (*n* = 326, *N* = 1,655,381).

^c^ Subpopulation is all included males (*n* = 248, *N* = 1,211,945).

d Patients who refused/not ascertained are not shown (*n* = 4 for worried, nervous, anxious, *n* = 5 for depressed).

## 3 RESULTS

### 3.1 Sample and population characteristics

During the third and fourth quarters of 2020, 574 (sample‐weighted estimate, 2,867,326) adults reported requiring cancer treatment and/or other cancer care since the start of the COVID‐19 pandemic. Of these, an estimated 24.7% reported receiving or supposed to be receiving cancer treatment only, including surgery, radiation therapy, chemotherapy, bone marrow transplants, stem cell transplants, or hormone therapy, while 47.8% reported only receiving or supposed to be receiving other care related to their cancer including lab visits, imaging, monitoring visits, rehabilitation, physical therapy, care for side‐effects, or visits with medical specialist. 27.5% reported requiring both. Table 1 shows the characteristics of all NHIS respondents requiring cancer treatment or other care related to their cancer during the COVID‐19 pandemic.

### 3.2 Changes, delays, or cancelation in treatment

**FIGURE 2:**
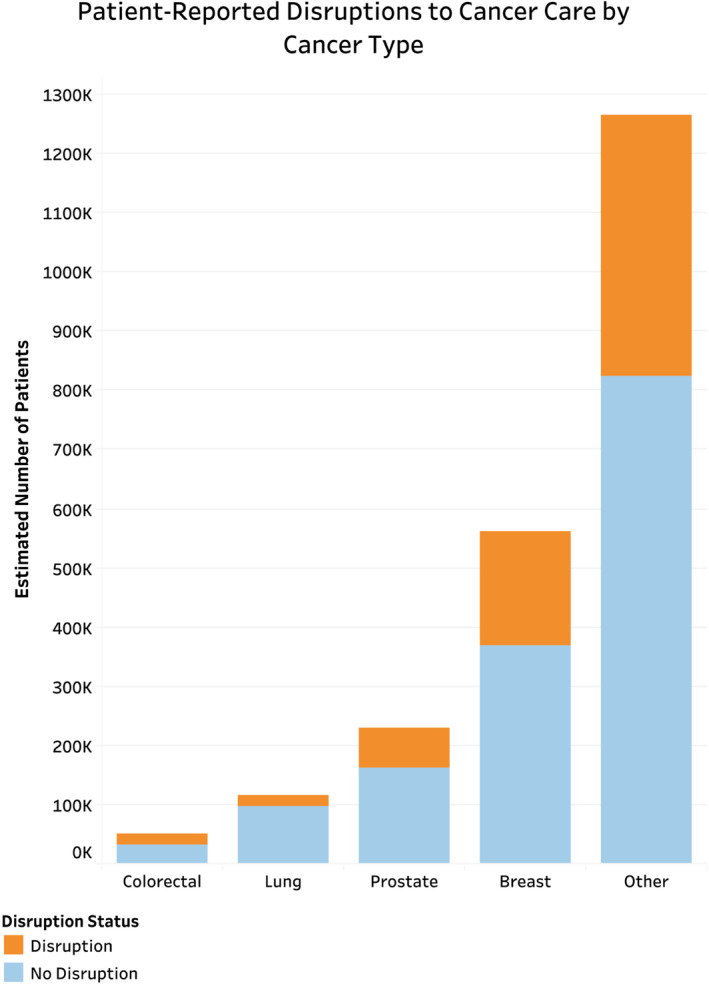
Patient‐reported disruptions in cancer care by cancer type for patients reporting history of one cancer. Patients reporting history of only one cancer were analyzed as this was the most accurate way of determining for which type of cancer patients were reportedly requiring treatment.

**FIGURE 3:**
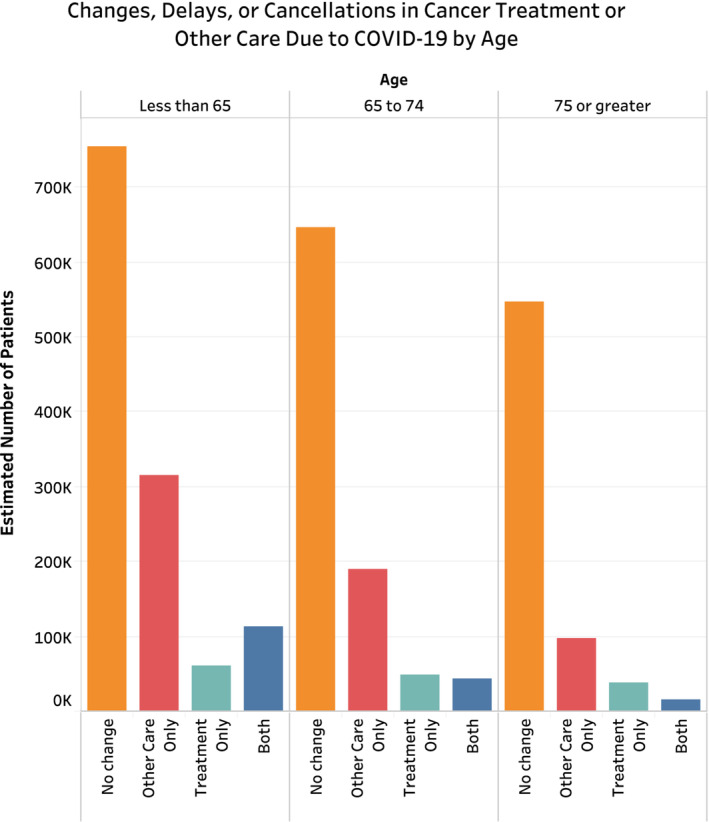
Changes, delays, or cancelations in cancer treatment or other care due the COVID‐19 pandemic stratified by age category.

Of the estimated 2,867,326 (*n* = 574) people reportedly requiring cancer treatment or other cancer care during the COVID‐19 pandemic, 32.1% reported any change, delay, or cancelation due to the pandemic, with 5.1% reporting changes in treatment only, 21.0% reporting changes to other cancer care only, and 6.0% reporting changes to both treatment and other cancer care (Table 2). On chi‐squared analysis comparing those with and without any disruptions to care, patients with disruptions in care were more likely to be younger (Figure 2), female (65.8% vs. 53.9%, *p* = 0.034), uninsured (8.0% vs. 1.4%, *p* = 0.011), less likely to have hypertension (41.1% vs. 55.6%, *p* = 0.016), less likely to have multiple comorbidities (38.8% vs. 52.4%, *p* = 0.011), and had higher rates of anxiety daily or weekly (43.1% vs. 25.3%, *p* < 0.001) (Table S1). Additionally, nearly 90% of patients reported virtual appointment use during the pandemic. There was no significant difference across racial/ethnic groups, urban–rural classification, region, household income, or immunosuppression status (Figure 3).

**TABLE 2:** Proportion of patients requiring cancer care or other treatment reporting disruptions due to the COVID‐19 pandemic

	*n* = 574	*N* = 2,867,326
Cancer treatment or other cancer care changed, delayed, or canceled due to Covid‐19		
No change	385 (67.1%)	1,946,405 (67.9%)
Treatment only	37 (6.4%)	147,562 (5.1%)
Other cancer care only	123 (21.4%)	600,940 (21.0%)
Both	29 (5.1%)	172,419 (6.0%)
Any change to treatment or other cancer care		
No	385 (67.1%)	1,946,405 (67.9%)
Yes	189 (32.9%)	920,921 (32.1%)

On sample‐weighted univariable analysis (Table S2), patients aged 75 or greater were significantly less likely than those less than 65 to experience disruptions to care (odds ratio [OR] 0.43 [0.23, 0.79]; *p* = 0.006). Females were significantly more likely to experience disruptions than their male counterparts (OR 1.64 [1.04, 2.61]; *p* = 0.035). Uninsured patients were also significantly more likely to experience disruptions to care than those with government‐sponsored insurance (OR 5.83 [1.33, 25.51]; *p* = 0.019). Patients with two or more comorbidities were significantly less likely to experience disruptions in care than those with zero or one (OR 0.58 [0.37, 0.91]; *p* = 0.017). Patients experiencing delays were also significantly more likely to experience feelings of worry, nervousness, or anxiety (OR 2.24 [1.43, 3.50]; *p* < 0.001). Race, urban–rural classification, region, household income, immunosuppression status and virtual appointment utilization had no significant association with care disruptions. Results of unweighted univariable analysis are demonstrated in Table S3, largely with no changes in conclusion regarding significance across variables. Specifically, the only changes found were uninsured status was not demonstrated significantly associated with change in cancer care, and patients were found to have increased association with daily or weekly depressive symptoms (OR 2.02 95% CI [1.26, 3.24], *p* = 0.004). Tables S4 and S5 contain further information regarding results of univariable logistic regression analysis for factors associated with changes to treatment and other cancer care, respectively.

On sample‐weighted multivariable analysis (Table 3), patients aged 75 or greater remained significantly less likely to experience any care disruptions (OR 0.43 [0.22, 0.84]; *p* = 0.013), whereas female patients remained significantly more likely to experience delays (OR 1.64 [1.03, 2.61]; *p* = 0.036). Insurance status, number of comorbidities, and other adjusted variables did not demonstrate significant associations. Results of unweighted multivariable analysis were also largely unchanged and are demonstrated in Table S6, with the only demonstrable significant changes being the effect size of associated increased risk with females decreased to just outside of significance (*p* = 0.07), while Black patients were found to have decreased associated risk of changes to cancer care (OR 0.36 95% CI [0.14, 0.94], *p* = 0.037).

**TABLE 3:** Sample‐weighted multivariable logistic regression analysis for any change to cancer treatment or other cancer care during the COVID‐19 pandemic

Characteristic	Odds ratio (95% CI)	*p*‐value
Age (years)		
< 65	Reference	—
65–74	0.75 (0.43, 1.30)	0.31
75 or greater	0.43 (0.22, 0.83)	0.014^a^
Sex		
Male	Reference	—
Female	1.64 (1.03, 2.61)	0.036^a^
Race		
White	Reference	—
Black	0.52 (0.18, 1.56)	0.25
Other	0.65 (0.29, 1.46)	0.78
Hispanic		
Yes	Reference	
No	1.61 (0.35, 7.42)	0.54
Urban–rural classification		
Large central metropolitan	Reference	—
Large fringe metropolitan	0.62 (0.33, 1.17)	0.14
Medium and small metropolitan	0.63 (0.35, 1.11)	0.11
Nonmetropolitan	0.86 (0.44, 1.70)	0.66
Household income		
Less than 40,000	Reference	—
40,000–79,999	0.92 (0.54, 1.58)	0.76
80,000 or greater	0.74 (0.44, 1.25)	0.26
Insurance status		
Government	Reference	—
Private	0.66 (0.41, 1.08)	0.10
Uninsured	3.61 (0.72, 18.0)	0.12
Number of comorbidities		
Zero or One	Reference	—
Two or more	0.68 (0.42, 1.11)	0.12

^a^ *p* < 0.05.

## 4 DISCUSSION

Utilizing nationally representative data from the NHIS 2020, our study characterizes the extent of oncologic care disruptions among patients during the COVID‐19 pandemic nationally. First, we demonstrated that nearly 1/3 (32.1%) of patients requiring cancer care during the pandemic reported changes, delays, or cancelations. Second, those requiring cancer care during the pandemic are a heterogenous group of individuals regarding age, sex, race, urban–rural classification, region, income, insurance status, comorbidities, immunocompromised status, and cancer diagnosis. However, we found that those who were uninsured, had one or fewer comorbidities, of female sex were significantly more likely to report disruptions of care while those ages 75 or older were significantly less likely on unadjusted analyses. On adjusted analysis, females were significantly (63%) more likely and those age 75 or greater were significantly (43%) less likely. Third, although nearly 30% of patients were immunosuppressed due to prescriptions (18.9%) and/or a health condition (20.1%), this did not significantly affect their access to care during the COVID‐19 pandemic. Fourth, nearly 90% of patients requiring cancer treatment during the pandemic had a virtual appointment due to COVID‐19. Fifth, patients experiencing disruptions were significantly (124%) more likely to report feelings of anxiety.

It is well known that the COVID‐19 pandemic and the resultant policy responses impacted healthcare delivery. Less described is how it impacted care delivery for cancer patients, who face unique challenges in accessing care. Prior to the COVID‐19 pandemic, prevalence of treatment delays in three common cancers, bladder, hepatocellular, and breast, in the US since the early 2000s have ranged from reports of 11%–15%, in stark contrast to the 32.1% of patients reporting delay in cancer care due to the COVID‐19 pandemic demonstrated in this study.^16^, ^17^, ^18^ A systematic review of global delays and disruptions in cancer care due to COVID‐19 demonstrated that physician or system‐related factors were the most frequently identified causes of disruptions in care. This was mainly due to reduction in service availability through personnel or other supply chain issues, and predominantly affected availability of medical visits, surgeries, procedures, radiotherapy and chemotherapy.^19^ Several adaptations were made in cancer care as per organizations of authority, contributing to changes and delays in care. For example, the American Society of Clinical Oncology (ASCO) published an editorial recommending adapting treatment regimens to reduce the number of patient visits, including use of oral chemotherapeutics over hospital‐based options, and larger intervals between dosing. They also suggested reducing treatment duration or delaying treatment in some cases.^20^

In addition, accredited cancer sites were asked to prospectively consider which patients could be safely delayed during what, at that time, was thought to be a limited number of months of COVID‐19 pandemic risk and develop guidelines based on these considerations. In our study, age less than 65 (compared with 75 or greater) and female sex, and presence of one or fewer comorbidities were all associated with significantly greater likelihood of reported disruptions to care in unadjusted analysis, with age less than 65 and female sex remaining significant predictors on adjusted analysis. This could be, in part, due to prospective delay in patients with more indolent cancers, such as low risk breast cancer patients, undergoing continuing care (e.g., with hormone therapy) falling into this category. Many of these patients would also fall into lower risk categories due to younger age and lesser burden of comorbidities. Older patients are more likely to experience severe COVID‐19, but are also to have more advanced disease, worse outcomes after diagnosis, and higher mortality rates making disruptions to cancer care potentially more costly.^21^, ^22^, ^23^ This does not, however, indicate that treatment was halted in these lower risk patients, as female patients, although significantly more likely to report disruptions in cancer care, were not significantly more likely to report disruptions to cancer treatment, an increasingly important distinction due to the negative effects associated with delays in treatment across certain cancers.^24^ Rather, disruptions in other cancer care were more common, which could be a reflection of risk stratification guidelines as mentioned, of which the impact on outcomes should be assessed longitudinally.

Black, White, or other race, and Hispanic ethnicity were not significantly associated with reported disruptions to care in our study. This finding is limited due to low rates of sampling of minority groups, despite adjustment for race in the NHIS weighting methods, described elsewhere. Our sample population includes 82.9% White and 6.4% Black race, and 6.4% Hispanic ethnicity when compared with the US population consisting of 61.6% White alone and 12.4% Black, and 18.7% Hispanic or Latino (Table 1).^15^, ^25^ Of note, unweighted analysis demonstrated decreased likelihood of Black patients to report delays in comparison to white patients, although these findings do not account for the inherent underrepresentation present in the sample, which is accounted for by sample‐weighting. Therefore, the study findings should be investigated further as racial disparities in access to care during the pandemic have been extensively documented elsewhere.^26^, ^27^

Results regarding treatment change, delay, or cancelation across cancer types are difficult to interpret as the impact of these disruptions may be variable across stage and cancer subtype. A model published in JAMA Oncology predicts that certain cancers favor delayed treatment while others favor immediate treatment. For example, the model predicted that treatment delays did not result in negative outcomes for prostate cancer patients, but they did for stage I‐III head and neck cancers.^28^ As mentioned previously, delays are also associated with differing effects on mortality rates across cancer types.^29^ Thus, the results of our study must be calibrated to the impact of disruptions of care for each cancer type and stage, as well as setting and patient characteristics. Furthermore, consideration of the impact of COVID‐19 on delay in diagnosis due to decreased rates of cancer screening leading to disruptions in care are not captured in this study, and the long term effects of these disruption to cancer diagnoses must be considered and further evaluated.^30^

Delays in cancer treatment have previously been shown to increase mortality across surgical, systemic, and radiotherapy indications for certain cancers.^29^ Cancer patients are more likely to experience severe COVID‐19 infection, poor hospital outcomes, and mortality.^9^, ^21^, ^31^, ^32^, ^33^ However, in determining continuation of chemotherapy and other immune‐suppressing therapies, a new risk vs benefit analysis must be performed: the risk of contracting COVID‐19 if treatment were to continue must be considered as there is large risk of transmission of infection at infusion centers. Jindal et al suggest that adjuvant chemo should be continued for early‐stage cancer with curative intent. For later stage cancers, there is a more complex decision at hand, that is dependent on the situation. The communication recommends that those with cancer of poor performance status, aggressive disease, or heavy tumor burden not receive chemotherapy.^34^

Approximately 30% of patients identified in this study self‐reported as immunocompromised status secondary to use of chemotherapy agents and/or health condition, making them vulnerable to more severe outcomes of COVID‐19.^35^, ^36^, ^37^ Despite this, immunocompromised status had no significant effect on patient‐reported disruptions. As each distinct cancer diagnosis carries its own unique challenges regarding immunosuppression and effects of delay on care COVID‐19 pandemic, further study of competing risks in these patients are necessary.

Additionally, we found that most cancer patients accessed care via telehealth during the pandemic (87.9%, weighted *N* 1,406,845). This finding is reflective of the rapid adaptation of telehealth options and coverage, and demonstrates the feasibility of telehealth as an option to increase future access to cancer care, especially in situations where barriers to in‐person care are present.^38^

Lastly, we found that patients experiencing disruptions to their cancer care were significantly more likely to experience feelings of anxiety, worry, or nervousness. The hidden toll of the pandemic has been the drastic increase in mental health disorders and burden. Based on our findings, patients experiencing disruptions to their care are at higher risk and must be counseled and provided appropriate resources to address these issues.

Limitations to our study include all limitations of the NHIS 2020 survey itself. These have been described elsewhere but include lower response rate of the 2020 survey due to telephone interviewing (48.9%) rather than in‐person interviewing, given social distancing measures, which could lead to non‐response bias, although sample weighting is designed to account for this. Additionally, the newly implemented 2020 questions designed to describe oncology patients' access to care only include two questions, inclusive of broad categories (care changed, delayed, or canceled), which does not allow for quantification of delay and relies on patient‐reported perceptions. Furthermore, the question does not specifically list immunotherapy or targeted therapies, which could exclude patients receiving solely these treatments. This study is also limited by selection bias, as patients may attribute delays due to other causes as due to the COVID‐19 pandemic itself, as well as survivor bias as the NHIS only samples living individuals, which could exclude patients who may or may not have experienced disruptions to care who did not survive the study period. There are several potential covariates that may have been modified due to the pandemic, that merit further study, including state‐specific data as states were differentially impacted by COVID‐19 both in terms of case rates and timing, as well as enacted policies and restrictions. The data are also limited in that it does not provide information on cancer subtype, stage, or type of treatment. Despite these limitations, our study's strength lies in its national characterization of disruptions during the COVID‐19 pandemic.

## 5 CONCLUSION

Approximately 1/3 of patients experienced disruptions to cancer care during the COVID‐19 pandemic. Patients with younger age or female sex were more likely to have disruptions in care, which may reflect risk stratification strategies in the early stages of the pandemic. Furthermore, these patients were significantly more likely to report anxiety. The longitudinal impact of disruptions in these groups on outcomes merits further study.

## AUTHOR CONTRIBUTIONS

**Jacob J Lang:** Conceptualization (lead); data curation (equal); formal analysis (lead); investigation (equal); methodology (lead); project administration (equal); resources (lead); software (lead); supervision (supporting); validation (equal); visualization (equal); writing – original draft (equal); writing – review and editing (equal). **Aparna Narendrula:** Conceptualization (supporting); data curation (equal); formal analysis (supporting); investigation (equal); methodology (supporting); project administration (equal); resources (equal); software (supporting); supervision (supporting); validation (supporting); visualization (supporting); writing – original draft (equal); writing – review and editing (equal). **Sharanya Iyer:** Investigation (equal); validation (equal); writing – original draft (equal); writing – review and editing (equal). **Kristine Zanotti:** Supervision (equal); validation (equal); writing – original draft (supporting); writing – review and editing (equal). **Puneet Sindhwani:** Conceptualization (supporting); investigation (equal); project administration (supporting); supervision (supporting); writing – original draft (supporting); writing – review and editing (equal). **Elias Mossialos:** Conceptualization (supporting); methodology (supporting); project administration (supporting); supervision (supporting); validation (supporting); visualization (supporting); writing – original draft (supporting); writing – review and editing (equal). **Obi Ekwenna:** Conceptualization (equal); investigation (equal); methodology (equal); project administration (equal); supervision (lead); validation (supporting); writing – original draft (equal); writing – review and editing (equal).

## CONFLICT OF INTEREST

None.

## Supporting information


Table S1

## Data Availability

The data underlying this article is publicly available at https://www.cdc.gov/nchs/nhis/2020nhis.htm.
