# Supplementary material for: Patient‐reported disruptions to cancer care during the COVID‐19 pandemic: A national cross‐sectional study
Source: Cancer Med. 2022 Oct 7;12(4):4773–85. doi: 10.1002/cam4.5270 (PMC9874402; doi:10.1002/cam4.5270)
Supplement: Supplementary file 1 — Table S1 [file CAM4-12-4773-s001.docx]

**Appendix A: Supplementary Tables**

**Supplementary Table 1**: Characteristics of patients reporting any disruptions to cancer care versus no disruptions due to the COVID-19 pandemic

| Characteristic | No change  (%) | Change  (%) | P-value |
| --- | --- | --- | --- |
| Age, y |  |  |  |
| Less than 65 | 38.8% | 53.6% |  |
| 65-74 | 33.2% | 30.5% | 0.016* |
| 75 or greater | 28.1% | 16.4% |  |
| Sex |  |  |  |
| Female | 53.9% | 65.8% | 0.034* |
| Male | 46.1% | 34.2% |  |
| Race |  |  |  |
| White | 82.6% | 83.6% | 0.64 |
| Black | 7.2% | 4.6% |  |
| Other | 10.3% | 11.8% |  |
| Hispanic |  |  |  |
| Yes | 6.7% | 5.9% | 0.80 |
| No | 93.3% | 94.1% |  |
| Urban-Rural Classification |  |  |  |
| Large central metropolitan | 22.9% | 27.6% |  |
| Large fringe metropolitan | 26.1% | 20.5% | 0.41 |
| Medium and small metropolitan | 33.0% | 30.1% |  |
| Nonmetropolitan | 18.0% | 21.8% |  |
| Region |  |  |  |
| Northeast | 19.0% | 15.8% |  |
| Midwest | 23.4% | 23.1% | 0.76 |
| South | 33.8% | 33.0% |  |
| West | 23.8% | 28.0% |  |
| Household Income |  |  |  |
| Less than 40,000 | 30.1% | 33.6% |  |
| 40,000-79,999 | 28.5% | 26.3% | 0.26 |
| 80,000 or greater | 41.5% | 40.1% |  |
| Insurance Status |  |  |  |
| Government Subsidized | 39.8% | 39.7% |  |
| Private | 58.8% | 52.3% | 0.011* |
| Uninsured | 1.4% | 8.0% |  |
| No. of Comorbidities |  |  |  |
| Zero or one | 47.6% | 61.2% | 0.016* |
| Two or more | 52.4% | 38.8% |  |
| Weakened Immune System due to Prescriptions |  |  |  |
| Yes | 17.3% | 23.0% | 0.15 |
| No | 82.7% | 77.0% |  |
| Weakened Immune System due to Health Condition |  |  |  |
| Yes | 20.3% | 20.9% | 0.90 |
| No | 79.7% | 79.1% |  |
| Worried, nervous, or anxious |  |  |  |
| Never, a few times a year, monthly | 74.7% | 56.9% | <.001* |
| Daily or weekly | 25.3% | 43.1% |  |
| Depressed |  |  |  |
| Never, a few times a year, monthly | 87.9% | 81.4% | 0.10 |
| Daily or weekly | 12.1% | 18.7% |  |
| Virtual appointment related to COVID-19 | (n=331) | (N=1,600,587) |  |
| Yes | 12.3% | 11.8% |  |
| No | 87.7% | 88.2% | 0.93 |
| Cancer subgroups^d^ | (n=436) | (N=2,222,992) |  |
| Breast | 65.7% | 34.3% |  |
| Prostate | 70.6% | 29.4% |  |
| Lung | 84.3% | 15.7% |  |
| Colon & Rectal | 63.8% | 36.2% |  |
| Other | 65.2% | 34.8% |  |

**Supplementary Table 2**: Sample-weightd univariable logistic regression analysis for any change to cancer treatment or other care during the COVID-19 pandemic

| Characteristic | Odds Ratio (95% CI) | P-Value |  |
| --- | --- | --- | --- |
| Age, y |  |  |  |
| Less than 65 | Reference | - |  |
| 65-74 | 0.67 (0.40, 1.14) | 0.14 |  |
| 75 or greater | 0.43 (0.23, 0.79) | 0.006* |  |
| Sex |  |  |  |
| Male | Reference | - |  |
| Female | 1.64 (1.04, 2.61) | 0.035* |  |
| Race |  |  |  |
| White | Reference | - |  |
| Black | 0.63 (0.24, 1.66) | 0.35 |  |
| Other | 1.14 (0.49, 2.66) | 0.77 |  |
| Hispanic |  |  |  |
| Yes | Reference | 0.80 |  |
| No | 1.15 (0.38, 3.46) |  |  |
| Urban-Rural Classification |  |  |  |
| Large central metropolitan | Reference | - |  |
| Large fringe metropolitan | 0.65 (0.36, 1.19) | 0.16 |  |
| Medium and small metropolitan | 0.76 (0.43, 1.32) | 0.33 |  |
| Nonmetropolitan | 1.01 (0.53, 1.90) | 0.98 |  |
| Region |  |  |  |
| Northeast | Reference | - |  |
| Midwest | 1.19 (0.62, 2.27) | 0.60 |  |
| South | 1.17 (0.64, 2.13) | 0.61 |  |
| West | 1.42 (0.74, 2.70) | 0.29 |  |
| Household Income |  |  |  |
| Less than 40,000 | Reference | - |  |
| 40,000-79,999 | 0.83 (0.48, 1.43) | 0.49 |  |
| 80,000 or greater | 0.87 (0.52, 1.45) | 0.58 |  |
| Insurance Status |  |  |  |
| Government | Reference | - |  |
| Private | 0.89 (0.57, 1.39) | 0.61 |  |
| Uninsured | 5.83 (1.33, 25.51) | 0.019* |  |
| Number of Comorbidities |  |  |  |
| Zero or One | Reference | - |  |
| Two or More | 0.58 (0.37, 0.91) | 0.017* |  |
| Weakened Immune System due to Prescriptions |  |  |  |
| Yes | Reference | - |  |
| No | 0.70 (0.43, 1.14) | 0.15 |  |
| Weakened Immune System due to Health Condition |  |  |  |
| Yes | Reference | - |  |
| No | 0.97 (0.58, 1.62) | 0.90 |  |
| Virtual appointment related to COVID-19 |  |  |  |
| Yes | Reference | - |  |
| No | 1.04 (0.44, 2.46) | 0.93 |  |
| Worried, nervous, or anxious |  |  |  |
| Never, a few times a year, monthly | Reference | - |  |
| Daily or weekly | 2.24 (1.43, 3.50) | <.001* |  |
| Depressed |  |  |  |
| Never, a few times a year, monthly | Reference | - |  |
| Daily or weekly | 1.67 (0.90, 3.12) | 0.11 |  |
| Cancer subgroups^d^ | (n=436) | (N=2,222,992) |  |
| Breast | 1.07 (0.57, 2.01) | 0.83 |  |
| Prostate | 0.82 (0.37, 1.81) | 0.63 |  |
| Lung | 0.36 (0.11, 1.18) | 0.09 |  |
| Colon & Rectal | 1.15 (0.26, 5.16) | 0.86 |  |
| Other | 1.19 (0.71, 2.00) | 0.52 |  |

^d^ – Reference is those without the specified cancer. Findings also non-significant with repeated analyses of each cancer used as reference group.

*Hispanic subgroups were omitted due to limited sample size

**Supplementary Table 3**: Unweighted univariable logistic regression analysis for any change to cancer treatment or other care during the COVID-19 pandemic

| Characteristic | Odds Ratio (95% CI) | P-Value |  |
| --- | --- | --- | --- |
| Age, y |  |  |  |
| Less than 65 | Reference | - |  |
| 65-74 | 0.73 (0.49, 1.10) | 0.14 |  |
| 75 or greater | 0.44 (0.28, 0.70) | 0.000* |  |
| Sex |  |  |  |
| Male | Reference | - |  |
| Female | 1.46 (1.02, 2.09) | 0.037* |  |
| Race |  |  |  |
| White | Reference | - |  |
| Black | 0.47 (0.19, 1.16) | 0.10 |  |
| Other | 0.87 (0.44, 1.72) | 0.70 |  |
| Hispanic |  |  |  |
| Yes | Reference | 0.69 |  |
| No | 1.20 (0.49, 2.95) |  |  |
| Urban-Rural Classification |  |  |  |
| Large central metropolitan | Reference | - |  |
| Large fringe metropolitan | 0.85 (0.52, 1.41) | 0.53 |  |
| Medium and small metropolitan | 0.83 (0.53, 1.31) | 0.42 |  |
| Nonmetropolitan | 0.89 (0.52, 1.51) | 0.67 |  |
| Region |  |  |  |
| Northeast | Reference | - |  |
| Midwest | 1.08 (0.62, 1.87) | 0.78 |  |
| South | 0.94 (0.55, 1.62) | 0.85 |  |
| West | 1.22 (0.71, 2.09) | 0.46 |  |
| Household Income |  |  |  |
| Less than 40,000 | Reference | - |  |
| 40,000-79,999 | 0.86 (0.56, 1.33) | 0.50 |  |
| 80,000 or greater | 0.93 (0.61, 1.40) | 0.71 |  |
| Insurance Status |  |  |  |
| Government | Reference | - |  |
| Private | 0.89 (0.68, 1.40) | 0.90 |  |
| Uninsured | 2.46 (0.73, 8.30) | 0.147 |  |
| Number of Comorbidities |  |  |  |
| Zero or One | Reference | - |  |
| Two or More | 0.65 (0.45, 0.93) | 0.017* |  |
| Weakened Immune System due to Prescriptions |  |  |  |
| Yes | Reference | - |  |
| No | 0.99 (0.64, 1.53) | 0.96 |  |
| Weakened Immune System due to Health Condition |  |  |  |
| Yes | Reference | - |  |
| No | 0.89 (0.58, 1.37) | 0.60 |  |
| Virtual appointment related to COVID-19 |  |  |  |
| Yes | Reference | - |  |
| No | 1.19 (0.60, 2.39) | 0.61 |  |
| Worried, nervous, or anxious |  |  |  |
| Never, a few times a year, monthly | Reference | - |  |
| Daily or weekly | 2.37 (1.63, 3.44) | <.001* |  |
| Depressed |  |  |  |
| Never, a few times a year, monthly | Reference | - |  |
| Daily or weekly | 2.02 (1.26, 3.24) | 0.004* |  |
| Cancer subgroups^d^ | (n=436) | (N=2,222,992) |  |
| Breast | 1.04 (0.65, 1.66) | 0.86 |  |
| Prostate | 0.65 (0.36, 1.27) | 0.21 |  |
| Lung | 0.59 (0.21, 1.66) | 0.32 |  |
| Colon & Rectal | 1.12 (0.32, 3.87) | 0.86 |  |
| Other | 1.24 (0.83, 1.86) | 0.29 |  |

**Supplementary Table 4**: Univariable logistic regression analysis for factors associated with changes, delays, or cancellation in cancer treatment only during COVID-19

| Characteristic | Odds Ratio (95% CI) | P-Value |
| --- | --- | --- |
| Age, y |  |  |
| Less than 65 | Reference | - |
| 65-74 | 0.58 (0.26, 1.32) | 0.19 |
| 75 or greater | 0.41 (0.18, 0.95) | 0.038* |
| Sex |  |  |
| Male | Reference | - |
| Female | 0.56 (0.27, 1.16) | 0.12 |
| Race |  |  |
| White | Reference | - |
| Black | 0.31 (0.62, 1.50) | 0.14 |
| Other | 0.54 (0.69, 4.27) | 0.56 |
| Urban-Rural Classification |  |  |
| Large central metropolitan | Reference | - |
| Large fringe metropolitan | 0.94 (0.37, 2.41) | 0.90 |
| Medium and small metropolitan | 1.07 (0.47, 2.42) | 0.87 |
| Nonmetropolitan | 1.42 (0.44, 4.63) | 0.56 |
| Region |  |  |
| Northeast | Reference | - |
| Midwest | 1.00 (0.32, 3.20) | 0.99 |
| South | 1.80 (0.68, 4.80) | 0.24 |
| West | 0.67 (0.24, 1.87) | 0.44 |
| Household Income |  |  |
| Less than 40,000 | Reference | - |
| 40,000-79,999 | 0.78 (0.33, 1.87) | 0.58 |
| 80,000 or greater | 0.96 (0.40, 2.28) | 0.93 |
| Insurance Status |  |  |
| Government Subsidized | Reference | - |
| Private | 1.56 (0.76, 3.19) | 0.22 |
| Uninsured | 42.96 (5.54, 333.21) | 0.000** |
| Weakened Immune System due to Prescriptions |  |  |
| Yes | Reference | - |
| No | 0.94 (0.47, 1.88) | 0.85 |
| Weakened Immune System due to Health Condition |  |  |
| Yes | Reference | - |
| No | 1.15 (0.52, 2.55) | 0.74 |
| No. of Comorbidities |  |  |
| Zero or one | Reference | - |
| Two or more | 0.51 |  |
| Virtual appointment related to COVID-19 |  |  |
| Yes | Reference | - |
| No | 0.95 (0.33, 2.71) | 0.92 |

**Supplementary Table 5**: Univariable logistic regression analysis for factors associated with changes, delays, or cancellation in other cancer care during COVID-19

| Characteristic | Odds Ratio (95% CI) | P-Value |
| --- | --- | --- |
| Age, y |  |  |
| Less than 65 | Reference | - |
| 65-74 | 0.68 (0.39, 1.21) | 0.19 |
| 75 or greater | 0.39 (0.19, 0.78) | 0.008* |
| Sex |  |  |
| Male | Reference | - |
| Female | 1.93 (1.15, 3.25) | 0.013* |
| Race |  |  |
| White | Reference | - |
| Black | 0.45 (0.14, 1.40) | 0.14 |
| Other | 1.31 (0.52, 3.27) | 0.56 |
| Hispanic |  |  |
| Yes | Reference | 0.87 |
| No | 0.92 (0.35, 2.40) |  |
| Urban-Rural Classification |  |  |
| Large central metropolitan | Reference | - |
| Large fringe metropolitan | 0.69 (0.35, 1.37) | 0.29 |
| Medium and small metropolitan | 0.67 (0.36, 1.27) | 0.22 |
| Nonmetropolitan | 1.10 (0.52, 2.33) | 0.80 |
| Region |  |  |
| Northeast | Reference | - |
| Midwest | 1.17 (0.57, 2.43) | 0.66 |
| South | 1.13 (0.57, 2.23) | 0.73 |
| West | 1.75 (0.82, 3.73) | 0.15 |
| Household Income |  |  |
| Less than 40,000 | Reference | - |
| 40,000-79,999 | 0.80 (0.43, 1.49) | 0.48 |
| 80,000 or greater | 0.97 (0.55, 1.72) | 0.93 |
| Insurance Status |  |  |
| Government Subsidized | Reference | - |
| Private | 0.82 (0.51, 1.32) | 0.41 |
| Uninsured | 5.84 (1.16, 29.48) | 0.033* |
| Weakened Immune System due to Prescriptions |  |  |
| Yes | Reference | - |
| No | 0.78 (0.44, 1.36) | 0.38 |
| Weakened Immune System due to Health Condition |  |  |
| Yes | Reference | - |
| No | 1.10 (0.61, 1.97) | 0.76 |
| Virtual appointment related to COVID-19 |  |  |
| Yes | Reference | - |
| No | 1.23 (0.39, 3.89) | 0.72 |

**Supplementary Table 6**: Unweighted multivariable logistic regression analysis for any change to cancer treatment or other cancer care during the COVID-19 pandemic

| Characteristic | Odds Ratio (95% CI) | P-Value |
| --- | --- | --- |
| Age, y |  |  |
| Less than 65 | Reference | - |
| 65-74 | 0.74 (0.47, 1.16) | 0.19 |
| 75 or greater | 0.42 (0.25, 0.70) | 0.001* |
| Sex |  |  |
| Male | Reference | - |
| Female | 1.42 (0.98, 2.06) | 0.07 |
| Race |  |  |
| White | Reference | - |
| Black | 0.36 (0.14, 0.94) | 0.037* |
| Other | 0.66 (0.23, 1.85) | 0.43 |
| Hispanic |  |  |
| Yes | Reference |  |
| No | 1.16 (0.30, 4.53) | 0.83 |
| Urban-Rural Classification |  |  |
| Large central metropolitan | Reference | - |
| Large fringe metropolitan | 0.80 (0.47, 1.34) | 0.39 |
| Medium and small metropolitan | 0.78 (0.49, 1.25) | 0.31 |
| Nonmetropolitan | 0.78 (0.45, 1.37) | 0.39 |
| Household Income |  |  |
| Less than 40,000 | Reference | - |
| 40,000-79,999 | 0.86 (0.54, 1.37) | 0.52 |
| 80,000 or greater | 0.79 (0.49, 1.27) | 0.34 |
| Insurance Status |  |  |
| Government | Reference | - |
| Private | 0.79 (0.52, 1.20) | 0.26 |
| Uninsured | 1.72 (0.47, 6.31) | 0.41 |
| Number of Comorbidities |  |  |
| Zero or One | Reference | - |
| Two or More | 0.75 (0.51, 1.09) | 0.13 |

**Appendix B: PubMed search terms used in background literature review**

(Oncology OR Cancer) AND (Delay OR disruption) AND (Covid OR Coronavirus)
